# Stigma as a barrier to early intervention among youth seeking mental health services in Ontario, Canada: a qualitative study

**DOI:** 10.1186/s12913-023-09075-6

**Published:** 2023-01-26

**Authors:** Natasha Y. Sheikhan, Jo L. Henderson, Tanya Halsall, Mardi Daley, Samantha Brownell, Jai Shah, Srividya N. Iyer, Lisa D. Hawke

**Affiliations:** 1 Centre for Addiction and Mental Health, 80 Workman Way, Toronto, ON M6J 1H4 Canada; 2 University of Ottawa Institute of Mental Health Research, 1145 Carling Avenue, Ottawa, ON K1Z 7K4 Canada; 3 Department of Psychiatry, McGill University, 1033 Pine Avenue West, Montreal, QC H3A 1A1 Canada

**Keywords:** Health services, Mental health, Stigma, Substance use, Youth

## Abstract

**Background:**

Stigma associated with mental health challenges is a major barrier to service seeking among youth. Understanding how stigma impacts service-seeking decisions from the perspectives of youth remains underexplored. Such research is necessary to inform effective stigma reduction.

**Objective:**

This study aims to understand how stigma influences service seeking among youth with mental health challenges.

**Methods:**

Qualitative inquiry was taken using youth engagement, underpinned by pragmatism. Data were collected via 4 virtual focus groups with 22 purposively selected youth participants with lived experience of mental health challenges in Ontario, Canada. Focus group guides were developed collaboratively among research team members, including youth co-researchers. Data were analyzed inductively using reflexive thematic analysis.

**Results:**

Three main themes were constructed from the data: point of entry into the system, being biomedicalized or trivialized, and paving the way for non-stigmatizing services. Initial contact with the mental healthcare system was seen to be affected by stigma, causing participants to delay contact or be refused services if they do not fit with an expected profile. Participants described a constant negotiation between feeling ‘sick enough’ and ‘not sick enough’ to receive services. Once participants accessed services, they perceived the biomedicalization or trivialization of their challenges to be driven by stigma. Lastly, participants reflected on changes needed to reduce stigma’s effects on seeking and obtaining services.

**Conclusion:**

A constant negotiation between being ‘sick enough’ or ‘not sick enough’ is a key component of stigma from the perspectives of youth. This tension influences youth decisions about whether to seek services, but also service provider decisions about whether to offer services. Building awareness around the invisibility of mental health challenges and the continuum of wellness to illness may help to break down stigma’s impact as a barrier to service seeking. Early intervention models of care that propose services across the spectrum of challenges may prevent the sense of stigma that deters youth from accessing and continuing to access services.

## Introduction

The stigma toward mental illness includes a variety of harmful stereotypical attitudes and behaviors enacted against people who have been labeled as mentally ill [1]. Common stigmatizing beliefs include the notion that mental illness signals personal deficits, weakness, difference, and a lack of self-control, and that people with mental illness cannot recover and are dangerous and violent [2]. Stigma intersects with culture and is found throughout all levels of society [3]—in the general public, within families and social circles, in the media and social media, among healthcare professionals, and among affected individuals themselves [4].

Stigma can be understood at three intersecting levels: structural, social, and self-stigma [5]. Structural stigma refers to the policies and practices of institutions that systematically restrict the rights and opportunities for people living with mental health disorders. Social stigma refers to the process whereby social groups endorse stereotypes about people with a stigmatized condition and act against them. Self-stigma occurs when individuals with mental health disorders internalize societal attitudes and discriminatory practices.

In 2018, some 10% of Canadian youth aged 12–24 years considered their mental health to be no better than “fair” or “poor” [6]. This proportion more than tripled in 2020, in the midst of the COVID-19 pandemic [7]. Youth are a critically important population in terms of mental health promotion, prevention, and treatment from a developmental perspective. About half of all mental health disorders first arise by about mid-adolescence [8]. Suicidal ideation and attempts are particularly high among youth [9]. The negative impacts of mental health challenges on developmental trajectories make adolescence an optimal time to intervene, both for full threshold mental disorders and subthreshold mental health challenges. There is also a need for greater investment in mental health promotion and prevention efforts [10]. Early intervention approaches, in particular, can connect youth with services before a full-threshold disorder develops. Although much work remains to be done in terms of developing effective, evidence-based early interventions for at-risk youth [11], such interventions have the potential to prevent medium- and long-term deterioration.

Despite the potential for intervening early, youth are highly impacted by stigma, and stigma can in turn constitute a major barrier to service seeking [12, 13]. In a systematic review, perceived stigma was found to be one of the main reasons youth choose not to seek mental health services [14]. Service seeking is fostered by factors such as parental support and social support, positive past service experiences, and the youth’s motivation to develop coping skills and address negative impacts of mental health challenges [13, 14]. A variety of mental health promotion and service design strategies have been recommended to foster service seeking among youth with mental health challenges, such as increasing health literacy [14], developing youth-friendly service settings that address a wide range of challenges [15, 16], and reducing stigma [17].

A range of stigma reduction approaches have also been examined [18]. For instance, intergroup contact theory proposes that direct contact with individuals impacted by stigma is key to reducing prejudice [19]. Although social contact has become a key ingredient of anti-stigma interventions, for providers, professional contact may not have the same stigma reduction effects [20]. Other approaches, including internet-based interventions [21], arts-based interventions [22], mass-media approaches [23], and peer support [24] also have potential. Among youth, classroom-based contact approaches have been used with some success [25]. If such interventions can reduce stigma, they may increase the willingness of youth to seek services when needed. However, the effect sizes of anti-stigma interventions are consistently small and stigma remains a considerable societal problem [18].

Researchers have been continually calling for more research on youth mental health stigma and stigma-reduction interventions to better understand the phenomena in various populations and reduce its negative impacts [26–28]. Since youth are embedded in families, schools, and societies, there are many potential sources of stigma and targets for stigma-reduction interventions. Identifying the mechanisms of how stigma influences decision-making may help guide those designing anti-stigma interventions on how to best break down stigma’s impact as a barrier to service seeking. The present study aims to understand how stigma influences service seeking decisions among youth with mental health challenges in Ontario, Canada.

## Method

### Design and setting

An experiential approach to qualitative inquiry was used to understand youth experiences and meanings [29]. Virtual focus group interviews were conducted in Ontario, Canada to elicit a rich understanding of the phenomena. The research team consisted of mental health researchers from the health and social sciences, clinicians, and youth co-researchers connected to a mental health and substance use clinical research setting. The present manuscript followed the Consolidated Criteria for Reporting Qualitative Research checklist [30].

### Epistemological stance

The present study was underpinned by a pragmatic worldview. As youth engagement occurred at all stages of this study, pragmatism was well suited to guide the study given the orientation towards real world practice and social change [31]. Pragmatism aligns with youth-oriented research as it is underpinned by democratic values, collaborative and action-oriented approaches, and social justice [31].

### Youth engagement

In accordance with the McCain Model of Youth Engagement [32], youth team members with lived experience contributed to all aspects of the project. Many youth members of the Centre for Addiction and Mental Health (CAMH) Youth Engagement Initiative have consistently identified stigma as an important research topic to pursue, guiding the initial project idea. Over the course of the project, five youth supported the research process through various roles, including three youth who were highly engaged in the project (one youth research analyst, one youth engagement specialist, and one youth data analyst), as well as two youth consultants who contributed on an advisory basis. Youth contributions included discussing the development of the grant application, co-developing study and recruitment materials, recruiting and consenting participants, co-facilitating the focus groups, analyzing and interpreting the data, and writing the manuscript. The three youth who contributed substantially to the project are co-authors (NYS, MD, SB).

### Participants

Participants included 22 youth with lived experience of mental health challenges. To be eligible, youth had to be aged 14–25 years, reside in Ontario, and self-describe as having experienced mental health challenges. Some participants had a pre-existing relationship with the research team as they had previously participated in research by the team and expressed interest in participating in future research.

### Procedures

Participants were purposively selected to increase sample diversity and breadth of data. In an initial open recruitment call, we sent a study flyer through the team’s networks of youth engagement entities and youth-serving organizations. We then examined several sociodemographic factors (age, gender, ethnicity) in the initial sample to identify gaps in diversity; accordingly, we used selective recruitment based on demographic characteristics to invite participants from a database of previous study participants in Ontario who consented to be contacted about future research. Potential participants contacted a research staff member by phone, email, or text, as per the study flyer, to learn more about the study. They were then invited to a screening and informed consent process, using the institutionally approved teleconferencing platform. Informed consent was collected electronically via REDCap software [33], after which participants provided demographic information on an unlinked REDCap survey. Participant numbers were assigned sequentially as participants were screened. Participants received a $50 honorarium. CAMH Research Ethics Board approval was obtained.

### Data collection

The research team conducted 4 semi-structured virtual focus groups from August to December 2021. A virtual platform was chosen for pragmatic reasons, given pandemic-related restrictions during the data collection period. The focus group discussions were approximately 120 min long, included 4 to 7 participants, and were held sequentially as participants were consented into the study.

The interview guide was developed collaboratively with the research team, including youth co-researchers. The guide included questions about the impact that various types of stigma have on service seeking, with a focus on attitudes, language, acceptance, discrimination, knowledge, and self-stigma. For instance, the guide included questions such as “When you’re struggling with mental health challenges, how does stigma affect the way you see your challenges?”, “Once you’ve made the decision to seek services, does mental health stigma make it harder to get those services?”, and “Once you’ve actually gotten into services, does mental health stigma affect how much you get involved or invested in the treatment?” The intersection of gender and other diversity factors with stigma was directly embedded in the interview guide, for example, “Do you think gender influences stigma? How does it influence your experience of stigma? How does it affect the way people feel or show stigma?” and “What other aspects of a person’s identity influence stigma?”.

Focus groups were co-facilitated by two youth with lived experience, one in a research analyst position and one as a youth engagement specialist. At the start of each focus group, they led a discussion about participants’ perspectives on stigma to help participants come to a common understanding of the topic. Discussions were recorded and transcribed verbatim. As focus groups were conducted virtually, participants had the option of using the chat function. To ensure that the chat content stimulated discussion among participants and was captured in the transcripts, facilitators read the content of the chat aloud as it occurred. A trained support worker was available if any participants needed extra support; this service was not used for any of the focus group discussions.

### Data analysis

Data were analyzed inductively using reflexive thematic analysis (TA) as outlined by Braun and Clarke [34]. Reflexive TA was chosen as it is a flexible approach to identify patterns and themes across the data. Reflexive TA is a recursive process and includes the following 6 phases: (1) data familiarization; (2) systematic coding; (3) generating the initial themes; (4) developing and reviewing the themes; (5) refining and defining the themes; and (6) writing the results. Within reflexive TA, themes are developed from codes and conceptualized as patterns of shared meaning. The analytic process was inductive and grounded in the data.

The coding process was conducted on NVivo 12 by a single analyst with lived experience (NYS). This process was organic and involved prolonged engagement with the data [35]. Throughout the analysis, ongoing meetings with NYS, LDH, MD, and SB were held to foster a rich nuanced interpretation of the data and enhance reflexivity [35]. For instance, the analyst (NYS) and lead (LDH) brought the tentative themes back to the youth co-authors (MD, SB) with representative quotes; through this discussion, they refined the themes, confirmed their relevance, and gained new interpretive insights. To aid quality practice, the analyst kept a reflexive journal throughout the research process. The written reflections were used to enhance self-awareness and reflect on researcher positionality.

## Results

Participants had a variety of backgrounds and characteristics (Table 1). Most participants had spoken to a professional about mental health or substance use. The analysis below discusses three main themes related to the influence of stigma on service seeking that were constructed from the data: (a) point of entry into the system (b) being biomedicalized or trivialized; and (c) paving the way for non-stigmatizing services.

**Table 1:** Demographic characteristics of participants

		Participants	
		(*n* = 22)	
Characteristic		n	%
Gender	Boy/man	7	31.8
	Girl/woman	10	45.5
	Transgender/non-binary	5	22.7
Age^a^	15–17	5	22.7
	18–25	17	77.3
Ethnicity	White	8	36.4
	East, Southeast, South Asian	6	27.3
	Black	2	9.1
	Indigenous	2	9.1
	Multiple or another ethnicity	4	18.2
First language	English	19	86.4
Area of residence	Large urban centre	15	68.2
Education	High school or less	9	40.9
	Some post-secondary	5	22.7
	Post-secondary diploma, degree, or certificate	8	36.4
Employment	Employed	18	81.8
Youth's self-rated mental health	Excellent, very good	2	9.1
	Good	9	40.9
	Fair/poor	11	50.0
Has spoken to a professional about mental health or substance use		18	81.8

^a^Participant age: mean = 21.3, standard deviation = 3.4

### Theme 1: Point of entry into the system

The first theme relates to how stigma shaped participants’ initial first contact with services. There are two subthemes: (a) delaying first contact; and (b) *‘am I sick enough?’*. This includes reasons for delaying first contact with services, and subsequently, having to be labeled as *‘sick enough’* to receive treatment.

#### Delaying first contact

Youth participants discussed various reasons for delaying first contact with mental health services. Their reasons related to limited literacy, shame, negative stereotypes, a fear of shifted identity, and label avoidance.

As participants reflected on why they delayed first contact, many believed they lacked appropriate knowledge and information about mental illness as it was not generally discussed. They recalled being unaware that their symptoms were related to mental illness. One youth noted how the shame associated with mental illness intersected with a lack of knowledge regarding their symptoms:

*I didn't realize that those symptoms were related to mental health and actually just carried a lot of shame with what I was going through or had a lot of self-blame for different things, and didn't even think of needing to access support just because you know… Versus when we're told about physical health like a stomach or headache going to a doctor, there's so many mental health symptoms that I didn't realize were something to seek support for. So, it was kind of that lack of education on top of the stigma of it.* [Focus Group 3]

Some discussed how negative stereotypes of people with mental illness contributed to inadequate knowledge about what mental illness looks like. This included gender stereotypes (for example, *‘boys don’t cry’*) and negative portrayals in the media (such as*, ‘dangerous’*). For instance, one youth mentioned, *“I honestly didn't think I had issues because they didn't match or seem the same as what I'd seen in the media.”* [Focus Group 2] These stereotypes contributed to feeling shame around wanting to seek services before first contact with the mental health system.

Youth participants also reported that they delayed seeking services as they were afraid service seeking would shift how they perceived themselves and how others might perceive them. One youth noted a dissonance between their ideal self and their self-image if they accessed services:

*I think that there’s a disparity between what, [I] wanted my image to be and then the perspective, if I access certain services, what that image would be…the self-stigma comes back where I’m putting myself into a certain box by accessing certain services and being open with that, which makes me less likely to want to use that.* [Focus Group 4]

Similarly, a few participants reported delaying seeking services because they felt that the negative stereotypes associated with mental illness would shift their professional identity, especially if they had to disclose. For example, one youth recalled feeling that a mental health diagnosis would jeopardize their career prospects:

*For me, I had some internalized stigma of when I was younger and thinking of my career goals and I did want to become a counselor. But then when I started realizing I needed support for my mental health, I had a lot of fear that if I did have a diagnosis of my own then I wouldn't be able to become a counselor, or, like, wouldn't be a good counselor and there would just be so much stigma attached to that.* [Focus Group 3]

A few participants reported avoiding treatment because of the negative labels they associated with medication. This included terms such as *‘antipsychotics’* and *‘antidepressants.’* One youth said, *“I refused to go on antipsychotics for so long because I was like, ‘No. I’m not crazy.’”* [Focus Group 4].

#### Am I sick enough?

After the initial barriers that delayed service seeking, youth reported feeling self-doubt about whether they were *‘sick enough’* for treatment. They explained that they were subsequently labeled by themselves and others as either *‘sick enough’* or *‘not sick enough’* to be treated. They expressed that *‘sick enough’* was often equated with being in crisis. Providers were seen as gatekeepers, holding the power to label youth as ‘sick enough’ for treatment.

Youth discussed self-doubt as a form of *‘imposter’s syndrome’* regarding whether they felt they were *‘sick enough’* to be using services. Imposter’s syndrome, as described by the participants, was when they felt they did not warrant the treatment and were taking away resources from. One youth noted:

*I know for me, one of the biggest barriers to my own mental health care was believing that I deserved [care]. [The concept of being sick enough] was what kept me from seeking treatment until I ended up needing in-patient services and things like that, that could have been avoided had I sought treatment earlier because I didn’t fit the stereotype of what someone who was “sick enough” looks like.* [Focus Group 4]

Youth recalled trivializing their own mental health concerns, such as *“I remember thinking my struggles weren't real and I was just ‘stupid’ because of how society sees it”* [Focus Group 2] and *“it becomes a lot of internalized questions of like, oh, am I sick enough?”* [Focus Group 4]. As they questioned whether they were *‘sick enough’*, some reported struggling with fitting into the narrow stereotypes for certain diagnoses.

As participants attempted to access services, they felt labeled as either *‘sick enough’* or *‘not sick enough’* by providers. The *‘not sick enough’* label represented a liminal space between sick and healthy, when youth did not fit into either category. Many described being denied treatment for not fitting into the stereotypical depiction of a mental health patient—this was a major barrier to early treatment. One stated that, *“there’s only one ‘look’ of people who can get that treatment early and it’s because they look like that stereotype — and if they don’t fit into that, you don’t get help”* [Focus Group 4]. Another described this process as humiliating:

*I don’t show up at 3 a.m. in the morning at [the hospital] because I want to put on a show for a social worker. I don’t. I don’t enjoy it, you know? I show up at 3 a.m. in the morning because I’m in crisis and I need help and I was hoping that finally I would get something. But to me it just feels like the stigma I face is like “Well, put on a good show and maybe we’ll help you if we have space, if we have the funding, or if we have room.” And it’s just – it’s really humiliating.* [Focus Group 1]

Participants felt they only received services when they were in a visible crisis, and as a result, could be labeled as *‘sick enough’*. For instance, a few participants who described eating disorders or eating-related symptoms recalled being turned away for not looking underweight enough, and subsequently, only receiving treatment once they lost even more weight. One participant was initially told *“You want to come in like a year?”* [Focus Group 4] because they were not *‘severely underweight’*, which they considered to be consistent with stereotypical depictions of eating disorders. Overall, youth reported that their symptoms were invalidated if they did not look sick enough.

### Theme 2: Being biomedicalized or trivialized

The second theme captures participant experiences in clinical settings and how they felt that their experiences were either trivialized or biomedicalized according to their perception of the application of the *‘medical model’*. It contains two subthemes: (a) ignoring the gray; and (b) symptoms are trivialized by providers. According to the participants, being biomedicalized included having to fit into reductive treatment approaches that included unnecessary labels, finite and structured options, and quick fix solutions. They perceived these approaches to be due to the valorization of the medical model by clinicians. Similar to the label *‘not sick enough’*, being trivialized pertained to being ignored or dismissed by providers during their care.

#### Ignoring the gray

Participants strongly criticized certain approaches to the care they received, citing them as a reflection of the medical model. Youth described a paternalistic and oversimplified approach that they considered to be inherent to the system. Several discussed feeling the gray areas of mental health were ignored by the service system: *“It’s like, you’re kind of given these black and white options and they don’t talk about gray areas.”* [Focus Group 4] The gray areas that participants felt were ignored pertained to treatment options and outcomes, both of which they felt were bound by the *‘medical model’* due to its perceived reductionist nature. Similarly, youth described services as being finite and oversimplified:

*We have a saying on campus, ‘It’s six sessions or less,’ because a lot of the staff will look at it and go, ‘You have six sessions. This can be fixed in six hours, essentially.’ When, in reality, it doesn't.* [Focus Group 2]

Many felt frustrated with how they were given a pre-determined number of sessions to feel better and were subsequently labeled as being either *‘healthy’* or *‘helpless’*. One participant expressed feeling discarded and blamed when they were labeled as *‘treatment resistant’*:

*Another attitude, and this is mainly on the part of clinicians I guess or the system, is like when they – how easily they label people ‘treatment resistant.’ Number one, it makes it really difficult to continue to like to receive any kind of support once you receive that label because you’re kind of just discarded, you know. Like, ‘okay, they’re just treatment resistant.’* [Focus Group 4]

Some discussed feelings of guilt, shame, and judgment for not feeling better after a set course of treatment. For example, one participant felt worried about the negative label they may receive if they did not get better:

*I felt getting judged by, like, friends or something, because like when you first get help they might be cheering you on, but if you're, like, in services for a long period of time, they might start thinking, like, you're crazy or something and say something about it. So I feel like that also, like, stops you from getting help to the full potential.* [Focus Group 3]

Another youth shared feeling shame and having to hide that they did not feeling better after six weeks of cognitive-behavioral therapy, stating that, *“I'm going to be seen as I wasn't trying hard enough.”* [Focus Group 2].

#### Symptoms are trivialized by providers

When youth did access services—despite the initial barriers—many felt their symptoms were trivialized by providers. Several described feeling dismissed and ignored. This was worsened when it intersected with stereotypes related to specific diagnoses, gender, and race.

A few participants indicated that trivialization and stigma differ by diagnosis. For instance, youth felt that the stigmatizing attitudes and stereotypes regarding schizophrenia were more severe and harmful compared to those about depression. Youth felt they were treated differently—and ultimately, trivialized—based on these attitudes. One participant referred to opioid use disorder as an example, stating that, from their experience, providers will dismiss any other symptoms they might have as due to opioid use, even if it is unrelated. Another felt that providers dismissed personality disorders as *‘unfixable’*. In varying ways, then, the youth felt that the stigmatizing attitudes they experienced from providers ultimately deterred youth from accessing services.

Some participants spoke of feeling dismissed and ignored as related to gender and race. A few felt that their providers minimized their symptoms as a normal experience for their gender (for example*,* provider saying, *“girls your age all have anxiety”).* In particular, trans youth expressed being faced with double stigma: *“As a trans person, I’ve had to prove myself as mentally ill and trans well.”* [Focus Group 4]. They felt that stigma manifested in their encounters with providers; they felt both blamed and dismissed:

*A lot of doctors do not know how to talk to trans patients, which leads to a lot of stigma. […] There’s still a lot of stigma going around especially with older therapists and older doctors that gender dysphoria is a mental health issue, because it was for a while and it was in the DSM. And I’ve had things that I’ve been going through blamed on my gender dysphoria and blamed on the dissatisfaction.* [Focus Group 4]

Trivialization also manifested through microaggressions for some racialized youth. For instance:

*The psychiatrist there pretty much just pinpointed all of my problems on like, ‘Oh your mother is too harsh of a parent.’ Really playing into those like tiger mom stereotypes and that completely – and you know just failed to provide any kind of services beyond that, and really demonized my family which is not helpful in the slightest.* [Focus Group 4]

Many participants indicated that trivialization contributed to the formation of negative perceptions regarding treatment. Ultimately, their difficult encounters discouraged them from accessing services again because they anticipated future experiences would be similar. In fitting with the previous sub-theme, youth tied their experience of feeling dismissed to stigma and *‘paternalistic attitudes’* in health care.

### Paving the way for non-stigmatizing services

To address the barriers to service seeking, the final theme captures several priorities for reducing stigma toward mental health services. This theme contains three subthemes: (a) shifting attitudes; (b) increasing awareness and dialogue; and (c) increasing service accessibility.

#### Shifting attitudes

Participants indicated the need to shift attitudes regarding mental health. This included valuing mental health like physical health, breaking down the dichotomy of mental illness versus wellness, and reducing stigmatizing forms of care. Many felt that treating and valuing mental health like physical health could reduce stigma. This was seen as an issue within and beyond the clinical setting:

*Viewing a diagnosis of mental illness in the same context that you would see a diagnosis of a physical one. So, in the same way that you wouldn’t blame someone with cancer for being, like, tired, or moody, you’re not going to blame someone with a mental illness for being tired and moody.* [Focus Group 4]

*One thing that can be done on kind of institutional level is to treat mental health the same way we treat other health. For example, when an employer provides benefits for health care or whatever... It’s always health and dental that's covered or have good coverage, or whatever. And mental health kind of is like an afterthought. […] Just acting, I guess, or showing that you accept mental health as an important part of health.* [Focus Group 2]

Reflecting similar frustrations with labeling in previous themes, a few participants discussed the need to shift attitudes regarding how mental health is traditionally approached. This includes transitioning from labels such as *‘sick’* versus *‘healthy’*, to viewing mental health on a continuum:

*I think a positive attitude is that it's, like, mental health is on a continuum and, like, sometimes thinking of it in a way that there isn't a fix and that there shouldn't be a fix. […] Maybe thinking of it as healthy versus normal. So, because we see mental health as so abnormal.* [Focus Group 3]

Lastly, a few participants mentioned the need to change certain aspects of care that perpetuate stigma. For instance, one youth stated that a starting point to reducing stigma includes *“changing the language we use, especially in a medical context, you know. Like, not phrasing things in ways that make people feel singled out or demonized.”* [Focus Group 4] This appeared to be a necessary change given that many participants had previously avoided services as a consequence of care that they experienced as stigmatizing.

#### Increasing awareness and dialogue

A desire for greater awareness and dialogue around mental health was widely expressed by participants. Many identified wanting greater open dialogue around mental health within their families and within institutions. For example, one youth discussed the need for open dialogue in schools to prevent instances where professionals may dismiss their mental health concerns:

*I think that with schools, like, talking about mental health openly would be really important, because guidance counsellors for me have pretty much always just been like ‘you are doing bad in science, that’s why you’re sad.’* [Focus Group 4]

Participants further discussed the need for increased mental health awareness in schools as early as possible. Some described education as a solution for combating stigmatizing attitudes, while others challenged this sentiment and indicated that education alone was not sufficient.

#### Increasing accessibility of services and accommodations

Participants discussed increasing access to mental health services and improving accommodations at school or work as solutions to mitigating stigma. Some described that addressing issues with the health system could in turn reduce stigma. For instance:

*Because we did talk about the long wait times and all the hoops sometimes to get services – addressing that on a larger scale so that it is easier to get service, and then in hopes reduces that stigma.* [Focus Group 3]

Lastly, a few participants suggested the need for greater accommodations in educational institutions or employers as one facet to reducing stigma. Reflecting prior discussions around treating mental health like physical health, one participant described ‘mental health days’ as a solution to reducing stigma:

*I think on a smaller level, having more opportunity for accommodations, whether it's at school or the workplace. And then maybe at a larger level having – I know some jobs have paid sick days, so paid mental health days as well that are equally as important and there's no stigma attached to it.* [Focus Group 3]

## Discussion

This study examined how stigma influences service seeking among youth with mental health challenges in Ontario, Canada. We generated three themes related to how youth may experience stigma during service delivery and their vision for future services: *point of entry into the system*, *being biomedicalized or trivialized*, and *paving the way for non-stigmatizing services*. Although self-stigma was initially a barrier to accessing mental health services, participants described that they must undergo a process of labelling to receive services. That is, youth can only receive services if they are deemed ‘sick enough’ by providers, in what they saw as an oversimplified classification of their lived experiences. During the labelling process, they expressed that their mental health challenges became either biomedicalized or trivialized. This deterred them from future services as they anticipated future encounters would be similar. Finally, participants described priorities for reducing stigma.

Previous research shows that stigma delays access to mental health services [28, 36, 37]. Consistent with existing literature on stigma among service-seeking youth [36, 38, 39], many youth in the present study delayed seeking services due to negative stereotypes that they encounter in the media, limited mental health literacy, label avoidance, and fear of shifted self-image. Moreover, disclosure-related concerns can also delay access to services [36]. In our study, disclosure concerns were raised by participants who felt that seeking services would shift their professional identity or jeopardize their career prospects. As most of the stigma research is carried out with adults [40], further research is needed on youth identity and mental health stigma as identity and career development contexts may differ for youth.

In clinical settings, it has previously been reported that people seeking mental health services often feel patronized, dismissed, and humiliated by their providers [41, 42]. Youth have reported that even providers minimize and belittle their mental health concerns, and that they are denied access to services if they are not considered sick enough [43]. Therapeutic pessimism—when providers hold pessimistic views regarding the likelihood of recovery—is also a source of stigma in clinical settings [42]. In the present study, participants referred to therapeutic pessimism as their challenges are considered ‘unfixable’ or they are quickly labeled as ‘treatment resistant’. Similar to our findings, Barney et al. [44] found that negative beliefs regarding symptom severity can shift the blame to individuals when they do not recover fully and quickly. In short, individuals would come to stigmatize themselves for their mental illness if they did not recover quickly and doubly if clinicians labeled them ‘unfixable’. However, most stigma research focuses on initial access to care [28]. Our findings highlight how clinical encounters that are experienced as stigmatizing also deter youth from accessing services at later points.

Stigma manifests across the spectrum of mental health challenges, negatively impacting life opportunities, quality of life, and self-esteem [45]. For milder mental health challenges, illness invalidation and controllability stigma emerge [45–47]. Illness invalidation stigma occurs when certain symptoms are viewed as trivial, while controllability stigma involves beliefs that individuals are responsible for resolving their symptoms on their own [47]. For example, in a recent study [48], youth reported invalidating experiences such as not feeling heard or seen by providers, being turned away from services, and non-recognition of the severity of their symptoms. Wrongful depathologization, such as when the severity of mental health problems is trivialized by providers, can increase stigma as it reinforces stereotypes that service users exaggerate their symptoms [49].

Many of our findings align with a conceptual model by Henshaw et al. [47] on treatment and illness stigma. Although the model is specific to depression, the components relate to the experiences of youth in our study. For instance, the model suggests that when people seek services soon after symptom onset, they are met with the stigma of being ‘not sick enough’ [47]. If they seek services too late, they are faced with the stigma of being ‘too sick’. Given the established value of early intervention [50], mitigating the stigma that prevents youth from accessing effective early interventions is a critical step to improving youth mental health.

Youth consider labels to be a double-edged sword. On the one hand, labels can facilitate help seeking for youth by enabling access to services, validating their experiences, and providing them with a greater understanding of their diagnosis [37]. On the other hand, labels place youth in a distinct illness category that can further exacerbate stigma. Although much of the literature focuses on the consequences of labelling as it relates to stigma [51], there are also consequences of withholding diagnostic labelling, for instance, placing youth in a ‘not sick enough’ category that disqualifies them from vital services. Moreover, oversimplified labels largely ignore the dimensionality aspect of mental illness. Lane [51] discusses the implications of taking a categorical understanding of diagnosis—similar to ‘sick enough’ vs. ‘not sick enough’—in terms of reducing access to services and resources. This is also shown in finite prognoses and treatment options as mental health problems are seen as acute, with a distinct endpoint [52]. Reflecting epistemic shifts in psychiatry, providers might consider moving away from categorical approach, towards a dimensional and transdiagnostic approach that cuts across traditional diagnostic boundaries and treatments [53]. This is especially needed for youth, as the complexities with emerging and early illnesses are difficult to capture with traditional taxonomies [54].

Early intervention models of care, such as transdiagnostic staging models that propose services for subthreshold symptoms [54], hold the potential to prevent negative mental health impacts by addressing symptoms earlier. Our findings suggest that such models might have the added advantage of reducing stigma and encouraging service-seeking behaviors, as they open their doors to youth who might elsewhere be deemed as ‘not sick enough’. With a focus on early symptom reduction and resilience building, wellness-based models have the potential to bring mental health out of a medical model that excludes them based on symptomatic thresholds—which, according to youth, drives stigma and poses a barrier to care. From this perspective, addressing youth mental health at the societal level requires a wellness focus that prioritizes health equity, strengthens protective factors, and supports positive youth development [55–57].

Despite increases in anti-stigma campaigns, stigmatizing attitudes held by providers remain [58]. In light of our findings, reducing stigmatizing forms of care should be prioritized in systems change. There is a need for patient-oriented research and service development co-designed with youth [16], in addition to anti-stigma training for providers early on in their careers [38, 39, 59]. Moreover, as a few participants reported their care being influenced by stereotypes related to gender, race, and transphobia, anti-oppressive pedagogies should be integrated into training programs. Racialized groups often face ‘double stigma’, with mental health stigma being an additional burden to prejudice, maltreatment, and discrimination as it relates to racism [60]. Future research should further explore youth experiences with ‘double stigma’, which remains largely unexplored for youth [36, 37].

### Strengths & limitations

This research demonstrates both strengths and limitations. A notable strength is the extent of youth engagement; engaging youth in all stages of the study enhanced the validity of the data and increased the relevance of the findings to the community. The remote nature of the focus groups enabled us to reach a more diverse sample and provided participants with an added means of contributing to the discussion, such as through the chat function; however, the virtual approach also excluded individuals without online access and prevented in-person group dynamics from occurring. Moreover, individual interviews may have evoked greater depth and clarity in the data compared to focus groups [61]. It is also important to note that these findings provide a snapshot of perspectives on stigma at a unique time in the history of global public health, given that the COVID-19 pandemic has had considerable repercussions for both youth mental health and associated services [62, 63]. Lastly, it is possible that the experiences reported are largely negative given that the interview guide did not include questions on positive experience with care. As suggested by MacDonald et al., future research on positive youth experiences with mental health services is needed to support best practices [39], in addition to exploring service provider views to gain a more fulsome picture.

## Conclusion

Stigma shapes the experiences of youth seeking mental health services, from delaying first contact with services to deterring youth from recurrent service use. Certain practices that place youth into ‘sick enough’ or ‘not sick enough’ categories warrant reconsideration—youth consider them an oversimplification of their lived experiences, which precludes many from accessing vital services and acts as a barrier to early intervention. Mitigating the stigma that prevents youth from accessing effective early interventions is a critical step to improving youth mental health. Stigma reduction initiatives would benefit from a dimensional approach that is inclusive of the mental health continuum, and targets both service users and providers.

## Data Availability

Data are available upon reasonable request to the corresponding author (lisa.hawke@camh.ca), with Research Ethics Board approval.

## Abbreviations

CAMH: Centre for Addiction and Mental Health
TA: Thematic Analysis
